# Perceptions of patients and providers on myocardial perfusion imaging for asymptomatic patients, choosing wisely, and professional liability

**DOI:** 10.1186/s12913-017-2510-y

**Published:** 2017-08-11

**Authors:** Kristopher P. Kline, Leslee Shaw, Rebecca J. Beyth, Jared Plumb, Linda Nguyen, Tianyao Huo, David E. Winchester

**Affiliations:** 1 Department of Medicine, College of Medicine, University of Florida, Gainesville, FL USA; 2 Department of Medicine, Emory Clinical Cardiovascular Research Institute, Division of Cardiology, Emory University School of Medicine, Atlanta, GA USA; 3 Geriatric Research Education and Clinical Centers, Malcom Randall VA Medical Center, and University of Florida Department of Medicine, Gainesville, FL USA; 4 Department of Medicine, College of Medicine, Division of Cardiovascular Medicine, University of Florida, 1600 SW Archer Road, PO Box 100277, Gainesville, FL 32610-0277 USA; 5 College of Medicine, University of Florida, Gainesville, FL USA; 6 Cardiology Section, Medical Service, Malcom Randall VA Medical Center, Gainesville, FL USA

**Keywords:** Myocardial perfusion imaging, Appropriate use criteria, Choosing wisely, Medical liability

## Abstract

**Background:**

Despite efforts by professional societies to reduce low value care, many reports indicate that unnecessary tests, such as nuclear myocardial perfusion imaging (MPI), are commonly used in contemporary practice. The degree to which lack of awareness and professional liability concerns drive these behaviors warrants further study. We sought to investigate patient and provider perceptions about MPI in asymptomatic patients, the Choosing Wisely (CW) campaign, and professional liability concerns.

**Methods:**

We administered an anonymous, paper-based survey with both discrete and open-response queries to subjects in multiple outpatient settings at our facilities. The survey was completed by 456 respondents including 342 patients and 114 physicians and advanced practice providers between May and August 2014. Our outcome was to compare patient and provider perceptions about MPI in asymptomatic patients and related factors.

**Results:**

Patients were more likely than providers to report that MPI was justified for asymptomatic patients (e.g. asymptomatic with family history of heart disease 75% versus 9.2%, *p* < 0.0001). In free responses to the question “What would be an inappropriate reason for MPI?” many responses echoed the goals of CW (for example, “If you don’t have symptoms”, “If the test is too risky”, “For screening or in asymptomatic patients”). A minority of providers were aware of CW while even fewer patients were aware (37.2% versus 2.7%, *p* < 0.0001). Over one third of providers (38.9%) admitted to ordering MPI out of concern for professional liability including 48.3% of VA affiliated providers.

**Conclusions:**

While some patients and providers are aware of the low value of MPI in patients without symptoms, others are enthusiastic to use it for a variety of scenarios. Concerns about professional liability likely contribute, even in the VA setting. Awareness of the Choosing Wisely campaign is low in both groups.

**Electronic supplementary material:**

The online version of this article (doi:10.1186/s12913-017-2510-y) contains supplementary material, which is available to authorized users.

## Background

Waste in medical spending is a widespread problem, estimated to be 30% of all health spending and equivalent to 750 billion US dollars annually [1, 2]. A variety of initiatives have been created by the medical profession to curb unnecessary spending, such as the Choosing Wisely (CW) campaign. This initiative was started by the American Board of Internal Medicine Foundation (ABIM) and asked medical societies to identify five things that patients and physicians should question. Lists created by multiple specialty societies cite overuse of nuclear cardiac stress testing or myocardial perfusion imaging (MPI) [3]. High rates of unnecessary MPI testing places burden on the medical system while exposing patients to unwarranted risks. In another initiative to reduce unnecessary and wasteful testing, professional societies have also developed Appropriate Use Criteria (AUC) to assist doctors in deciding which patients may benefit from MPI in common clinical scenarios [4].

These efforts are laudable, but their acceptance and implementation by front line providers in clinical care is unknown. Furthermore, the role of patients in these processes is limited, or nonexistent; patients’ reflections on appropriateness and awareness of waste and liability risks have not been studied. To investigate these issues, we conducted a survey of patients and healthcare providers asking a variety of questions related to these issues. We hypothesized that patients would be more likely than providers to endorse the use of MPI in the absence of symptoms.

## Methods

### Survey questionnaire

We developed and administered an anonymous survey to patients who have had MPI or had one ordered for them in primary care clinics, cardiology clinics, and nuclear medicine laboratories at both our academic medical center and affiliated Veterans Affairs (VA) medical center. We developed another survey with a parallel structure and administered it to healthcare providers, sampled from physicians and advanced providers (nurse practitioners and physician assistants) within primary care, cardiology, and hospital medicine. Providers were from within the same academic medical center. Its affiliated VA medical center, and private practice. When appropriate, identical questions were asked of both patients and providers.

**Fig. 1:**
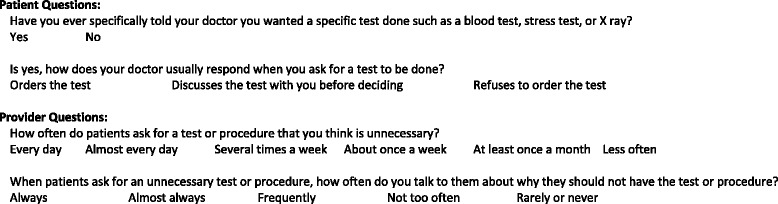
Sample questions showing parallel structure as reported to patient respondents versus provider respondents

Respondents provided demographic information and answered a wide variety of questions relating to the CW campaign, professional liability, unnecessary MPI, and wasted medical spending. Patients and providers were asked questions to gauge understanding on risks versus benefits of MPI testing. Survey questions were in a variety of formats including yes/no, scale responses, multiple choice, and narrative/free response. (For sample question please refer to Fig. 1; full questionnaire available as Additional files 1 and 2) Patient and provider attitudes towards AUC for MPI from this survey have been previously reported; these survey questions and those for asymptomatic testing were developed from previous studies on inappropriate MPI. [5–7] Survey questions for CW, professional liability and wasted medical spending were developed by authors to investigate potential contributing factors.

### Survey administration

The study was approved by our Institutional Review Board. Surveys were administered in paper form between May and August 2014. Informed consent was obtained, however the requirement for written documentation of informed consent was waived in order to maintain anonymity for all participants. Patient participants were recruited by study personnel (KK, JP, LN, DW) at patient check-in for appointments at a variety of clinical locations as noted above. Blank forms were also made available at appointment check-in to be completed voluntarily. Providers within our academic medical center and affiliated VA medical center and recruited through staff meetings; providers from private practice were recruited at statewide professional meetings and through personal interactions. Because of this methodology, and because regulatory oversight authorities did not permit formal tracking of those who were approached but did not elect to participate, an accurate denominator to calculate the percent responding to the survey cannot be provided. Results from completed surveys were entered into the secure, web-based application Research Electronic Data Capture (REDCap) database (authors JP, KK, and LN) [8].

### Statistical analysis

Baseline characteristics and survey responses were compared by ANOVA, Chi-square test, Fisher’s exact test, or Mantel-Haenszel test as appropriate for continuous, categorical, or ordinal variables as appropriate. Statistical calculations were performed on SAS version 9.4 (SAS Institute, Cary NC).

## Results

The survey was completed by 456 respondents, 342 patients and 114 providers. Three hundred fourty two patient respondents were distributed equally between cardiology (32.2%), primary care (36.0%), and nuclear medicine (31.9%) clinics; 55.6% were Veterans. (Table 1) Patient respondents were predominantly male (68%), and finished at least some college education (67.5%). Providers were from academic practice (61%), VA practice (27%), or private practice (10%). The majority were physicians (87%) followed by advanced care providers (13%). Primary specialty was cardiology (35.1%), primary care (37.7%), hospital medicine (22.8%), and other (4.4%).

**Table 1:** Patient and provider baseline characteristics

	Total		Total
Patients	*n* = 342	Providers	*n* = 114
Gender (male)	232 (68%)	Professional Training	
Age	61 ± 14	MD/DO	99 (87%)
Prior myocardial infarction	62 (18%)	ARNP/PA	15 (13%)
Prior PCI	56 (16%)		
Prior CABG	48 (14%)	Level of Experience	
Diabetes	126 (37%)	In training	48 (42%)
High blood pressure	236 (69%)	<5 years	20 (17.5%)
High cholesterol	180 (53%)	5–15 years	23 (20%)
Education (all responses)		>15 years	23 (20%)
Did not finish high school	19 (6%)		
Finished high school	87 (26%)	Primary specialty	
Some college classes	118 (35%)	Primary Care	43 (38%)
Graduated college	69 (20%)	Hospital Medicine	26 (23%)
Graduate or professional school	44 (13%)	Cardiology	40 (35%)
		Other	5 (4%)
		Primary workplace	
		Academic	71 (62%)
		VA	31 (27%)
		Private	9 (7%)
		Other academic	3 (3%)

### MPI in asymptomatic patients

Patients and providers were asked to consider a patient without symptoms and in what situations it would be appropriate to perform MPI, results are shown in Table 2. 75% (*n* = 238) of patients believed a family history of coronary artery disease warranted an MPI compared to just 9.2% (*n* = 10) of providers. 33% (*n* = 96) of patients considered having erectile dysfunction an indication for an MPI, while just 3.6% (*n* = 4) of providers agreed with this statement. Only 1 (0.9%) provider thought annual evaluation with MPI for asymptomatic patients was indicated, compared to almost a quarter (23%, *n* = 71) of patients.

**Table 2:** Patient and provider opinions on what justifies an MPI in asymptomatic patients

Which of the following justify an MPI?	Patient	Provider	*p* value	OR	95% CI
Diabetes mellitus	102 (35%)	28 (25.7%)	0.093	1.55	0.92–2.61
ECG changes	263 (86%)	63 (57.3%)	<0.0001	4.56	2.70–7.73
Family history of CAD	238 (75%)	10 (9.2%)	<0.0001	30.21	14.43–64.95
Erectile dysfunction	96 (33%)	4 (3.6%)	<0.0001	12.91	4.42–42.47
High risk of heart disease	272 (86%)	48 (44.4%)	<0.0001	7.73	4.57–13.09
Annual evaluation	71 (23%)	1 (0.9%)	<0.0001	32.79	4.8–643.97
Preoperative evaluation for low-risk surgery	67 (22%)	3 (2.7%)	<0.0001	10.08	2.97–41.09
Prior history of MI 1 year prior	246 (80%)	16 (14.5%)	<0.0001	22.94	12.18–43.76
Prior history of stents 1 year prior	209 (69%)	15 (13.6%)	<0.0001	13.93	7.4–26.51

### Unnecessary testing

We asked respondents to detail their experience with patient driven requests for specific testing. When we asked patients if they have ever specifically asked their provider to order a test, 40% responded that they had. twenty five reported that the provider orders the test without any discussion or inquiry as to why the patient wanted the study. The most common reasons patients reported requesting a test were family history of disease (23%), risk factors (16%), or at the recommendation of a family member (4%). Only 3 respondents (1%) requested a study because they heard about it on television or the internet. Among providers, 13.3% reported that patients requested unnecessary tests every day in their practice; 23.9% indicated this occurs almost daily. Among providers receiving frequent requests for unnecessary testing, 44.3% were academic, 33% private and 17.2% VA (*p* = 0.034).

Both providers and patients were asked to reflect on reasons why testing might be inappropriate. (Table 3) Many of the provider responses could be matched to an inappropriate MPI indication while some responses, such as liability concern and patient/family demand is not specifically noted in the AUC. Providers indicated that 31% of them have ordered a MPI only because the patient demanded it. Patient and provider narrative responses by rank and their correlation to published AUC are shown in Table 3.

**Table 3:** Patient and provider narrative responses by rank and their correlation to published AUC

Patients: Inappropriate Reasons for MPI (*n* = 149)	2009 AUC	Number
If the test is too risky	N/A	11
If you do not have symptoms	#7–11	10
If the test is not necessary	N/A	10
If the radiation risk is high	N/A	6
The doctor should decide	N/A	6
If the cost is too high	N/A	1
False positive testing	N/A	0
Providers: Inappropriate reasons for MPI	2009 AUC	Number
Asymptomatic	#7–11	26
Patient or family demand	N/A	16
Concern about professional liability	N/A	12
Screening in low risk patients	#7	9
Annual MPI	#45–56, #66–70	8
Preoperative risk assessment	#71	8
Symptoms with a normal ECG and can exercise	#1	8
Low risk chest pain	#1–2	6
Acute coronary syndrome	#10	6
Repeat test, no change in symptoms	#45–56, #66–70	5
Financial gain	N/A	4
Risk factors/Family History/Abnormal ECG alone	N/A	4
Chest pain with high pretest likelihood	#5–6	3

### Choosing wisely

37.2% (*n* = 42) of providers had heard of the CW campaign. Of the providers who were aware of CW a greater percentage were academic (48.6%) compared to private practice (11.1%) or the VA (20.7%) (*p* = 0.008). Proportionally more providers in training had heard of CW (44%) compared to providers <5 years from training (19.5%), 5–15 years from training (14.6%) or >15 years from training (9%) though this was not statistically significant (*p* = 0.59). Patients were less likely than providers to be aware of the CW campaign (2.7% versus 37.2%, *p* < 0.0001). In narrative responses, providers accurately described goals of the CW campaign, including appropriate testing recommendations, reduced waste spending, professional society based guidelines, and encouragement of shared decision making. Of the less than 3% of patients who had heard of the campaign, no narrative responses were reported describing the campaign’s goals.

### Liability risks

The majority of providers (65.2%) felt their employment provided good or excellent professional liability protection; this proportion was significantly lower for private practice providers (33.3%) compared to university affiliated (73.9%) and VA providers (55.2%) (*p* = 0.027). Providers remain concerned about professional liability with 15.9% of respondents stating they were very concerned, 36.3% concerned, and 35.4% being slightly concerned. Only 12.4% reported being not concerned over professional liability. More than a third (38.9%) of providers admitted that they had ordered a MPI in the past strictly out of concern for liability risk if they failed to do so.

### Waste and cost

We created questions based on the cost of MPI, published estimates of the total cost (in US dollars), and the percentage of US healthcare spending that is wasted to determine if patients and providers had accurate assessments of these facts. Compared to patients, providers more frequently identified the correct total cost (20.9% versus 10.4%, *p* < 0.0001) and percentage of costs (77.5% versus 39.3%, *p* < 0.0001) of wasteful spending. Over half of providers (53.7%) and patients (60%) correctly estimated the cost of a nuclear stress test to be >$1000.

### Radiation and cancer risks

In addressing risks of MPI, we asked questions about radiation exposure and understandings about radiation sources and risk. Two-thirds of patients correctly recognized that CT scans (65%) and nuclear stress tests (67%) were sources of radiation while 67% reported microwaves and 46% reported televisions were radiation sources. When asked to identify which entity had the most radiation exposure the top results were CT scans (33%), nuclear stress tests (32%) followed by microwaves (18%). Close to half (42%) of patient respondents reported there was no increased risk of cancer after getting MPI.

## Discussion

We performed a large survey of both patients and providers taken predominantly from a single academic health center and affiliated VA hospital system to gauge opinions and attitudes about MPI, appropriateness, and a variety of related topics. The results of the survey indicate areas for improvement in understanding about MPI and provide some striking observations about unnecessary testing and professional liability.

Patient respondents to our survey were more likely to report that MPI was warranted in a variety of clinical scenarios for asymptomatic patients. This is in accord with a recent comprehensive systematic review that showed patients tend towards overestimating benefits while underestimating harm regardless of the intervention [9]. Patients are also poorly informed by their providers on the risks of over-diagnosis and overtreatment, as shown in a survey which noted only 10% of individuals were explained these risks when undergoing cancer screenings [10]. Contributing to information mismatch is the fact that providers are more likely to explain the pros of testing and treatments instead of the potential cons, and patients often do not feel involved in shared decision making [11–13]. Informed-decision making strategies via balanced discussions between patients and providers are key to improving these disparities.

In free-text responses about inappropriate tests, both providers and patients offered many accurate reasons why testing is unnecessary. Providers gave responses that matched a number of the AUC for MPI. Both also gave responses that indicated ongoing concerns and awareness of professional liability. Not surprisingly, our data contributes to the literature that physicians are less concerned about professional liability when they are employed by an entity with sovereign immunity [14, 15]. We are concerned by the observation that over one third of providers openly admitted to ordering MPI specifically due to professional liability concerns. Similar findings were reported in a 2011 survey of primary care physicians showed that 76% ordered more aggressive testing due to liability concerns. [16] Another recent survey by an independent group for the American Board of Internal Medicine Foundation Choosing Wisely campaign reported that 52% of physicians felt the major reason for ordering unnecessary testing was professional liability concerns [17]. We observed among narrative responses from providers, liability concerns was the third most common “inappropriate” indication for ordering MPI behind asymptomatic patients and patient/family demand. In the same CW survey, 53% of ordering providers stated they would order an unnecessary test if a patient was insistent compared to 31% in our survey. Recent data suggest that “demanding” patients, those who go a clinical encounter with a specific request for a test or procedure, typically uncommon. Only 9% of encounters in outpatient oncology centers had such patients and that typically providers agreed with the patients’ concerns [18]. Only 1% of these encounters were deemed to have a clinically inappropriate patient demand and that these were rarely fulfilled. While patient satisfaction appears to improve with increasing knowledge about their conditions, medications and procedures, some tests and procedures may be limited in their opportunity for patient involvement in decision-making [19]. The core of the CW campaign and patient-centered medicine is to respond to “demanding” patients with respectful understanding and communication about their concerns [20].

While a proportion of providers in our survey were aware of CW (37.2%), we noted this was higher than national averages based on CW random polling data (21%) [17]. We suspect this is secondary to our study being performed at an academic medical institution. Awareness of the campaign among patients was only 2.7% in our study as opposed to about 5% as a national average. This demonstrates a continued disparity between patient and provider knowledge of the campaign and low awareness of the campaign overall.

Respondents to our survey had modest awareness of waste in the US healthcare system and efforts to reduce it. Patients were less likely than providers to accurately estimate the amount of waste, both measured in US dollars and as a proportion of total spending. Polled physicians feel aware of costs for tests/treatments and think providers should adhere to guidelines that discourage marginally beneficial care [21]. An overwhelming 89% agreed that doctors should play a bigger part in reducing unnecessary tests. A recent survey by Colla et al. showed nearly all physicians felt a responsibility to limit unnecessary testing, control costs, and adhere to guideline recommendations (96.8%, 92.2%, and 97.9% respectively) [22]. Another important finding in our survey is an estimate of the proportion of “rarely appropriate” tests that would be acceptable. The Institute of Medicine has shown that over a quarter of the $750 billion dollar annual cost of waste in health care is directly attributed to unnecessary services [2]. Currently, there is no agreement on what an acceptable rate of unnecessary services should be, but the most common response to this in our survey suggests a goal of less than 5% would be reasonable [7].

Patient responses also indicated a poor understanding of radiation exposure with 42% of respondents stating “no radiation exposure” associated with nuclear testing and 46% reported a “slight increase of exposure”. This is similar to a recent survey showing that 85% of patients undergoing CT or cardiac SPECT imaging underestimated the amount of radiation and 88% were not worried about scan radiation [23]. Another survey noted only 3% of patients had been informed about radiation risks prior to their last imaging study [24]. This is a major concern when its estimated that greater than 10% of the ionizing radiation exposure in the U.S. can be directly attributed to nuclear stress testing [25]. Furthermore, providers are also often misinformed about radiation exposure [26]. A 2011 survey of American Society of Nuclear Cardiology members showed only 9% could accurately estimate the radiation exposure of the most common nuclear stress test protocol [27]. Real time feedback has been proposed to assist with this problems as one recent study showed that displaying radiation exposure risks and imaging test costs at the time of ordering influenced providers on test appropriateness and helped facilitate physician-patient discussions [28]. More research is necessary to better guide informed decision-making for both patients and providers on radiation risks.

Our investigation has important limitations to acknowledge. The survey was conducted at a single site and may not be representative of broader populations. The methods used for population sampling may bias results towards a more favorable view of MPI. Responses were voluntary and rate of returned surveys were not tracked per Institutional Review Board regulations, leaving potential for bias due to absent responses. This is offset by the anonymous nature of the survey, especially regarding questions about professional liability and unnecessary testing. Survey responses by providers may not represent their actual clinical decision-making and ordering habits due to social desirability bias.

## Conclusion

While efforts such as Choosing Wisely strive to improve communication and reduce low-value care, awareness and implementation of the campaign in the community remains low. Liability concerns and patient demand may still contribute to test overuse and ordering in rarely appropriate circumstances. Providers reported low rates of using AUC and had concerns about their purported benefits. Both patients and healthcare providers underestimate the costs of wasteful medical spending, patients to a greater degree. These findings suggest that further study and resources are needed to encourage patients and providers to adopt cost-conscious and appropriate medical testing.

## Additional files

Additional file 1: Patient Survey. Description: Full version of questionnaire given to patients. (DOCX 25 kb)

Additional file 2: Provider Survey. Description: Full version of questionnaire given to providers. (DOCX 27 kb)

## Abbreviations

AUC: Appropriate Use Criteria
CW: Choosing Wisely
MPI: Myocardial perfusion imaging
VA: Veterans Affairs
