# Supplementary material for: Perceptions of patients and providers on myocardial perfusion imaging for asymptomatic patients, choosing wisely, and professional liability
Source: BMC Health Serv Res. 2017 Aug 11;17:553. doi: 10.1186/s12913-017-2510-y (PMC5553740; doi:10.1186/s12913-017-2510-y)
Supplement: Supplementary file 1 — Patient Survey. Description: Full version of questionnaire given to patients. (DOCX 25 kb) [file 12913_2017_2510_MOESM1_ESM.docx]

**1. Where are you being treated today?**

Malcom Randall VA

University of Florida

**2. Which type of clinic are you visiting?**

Primary Care

Cardiology

Nuclear laboratory

**3. Were you having chest pain symptoms when your physician ordered the nuclear stress test?**

Yes

No

**4. Please circle which clinic you are currently visiting:**

Rural (Starke, Old Town, VA CBOC)

Non rural (UF, Gainesville, Lake City)

**5. Please select your gender**

Male

Female

**6. Your Age**: _______________ (if over 89, just check here )

**7. Do you have or have you ever had any of the following medical problems? (circle all that apply)**

Heart attack

Cardiac stent

Bypass surgery

Diabetes

High blood pressure

High cholesterol

**8. What is the highest level of education you completed? (circle one)**

Did not finish high school

Finished high school

Some college classes

Graduated college

Graduate or professional school

**9. What kind of insurance do you have? (circle all that apply)**

None

Veterans Affairs coverage (including disability or service connection)

Private insurance

Medicaid

Medicare

**10. How often do you have someone help you read hospital materials?**

None of the time

A little of the time

Some of the time

Most of the time

All of the time

**11. How often do you have problems learning about your medical condition because of difficulty reading hospital materials?**

None of the time

A little of the time

Some of the time

Most of the time

All of the time

**12. How confident are you filling out forms yourself?**

All of the time

Most of the time

Some of the time

A little of the time

None of the time

**Read each story and think about whether the person should have a nuclear stress test of the heart. On a scale that goes from 1 (bad idea) to 9 (good idea), tell us what you think about doing a stress test. Circle one number for each story.**

13. A 62 year old woman had a heart attack 2 years ago and got a stent to open the artery. Recently, she had chest pressure when walking with her friends. She also has high blood pressure, high cholesterol and diabetes and she takes several pills for her heart.

Bad Idea | Okay Idea | Good Idea

1 2 3 4 5 6 7 8 9

14. A 45 year old woman has tightness in her chest that happens sometimes when she is carrying groceries in from the store and lasts a couple of seconds. She does not have any heart problems and can walk without any trouble.

Bad Idea | Okay Idea | Good Idea

1 2 3 4 5 6 7 8 9

15. A 52 year old man is worried because his father died at 52 of a heart attack. He feels fine, smokes a pack a day of cigarettes and does not have any other health problems.

Bad Idea | Okay Idea | Good Idea

1 2 3 4 5 6 7 8 9

16. A 58 year old woman had some chest pain while climbing stairs, but it also happened when she was watching TV. The pain gets better when she rests.

Bad Idea | Okay Idea | Good Idea

1 2 3 4 5 6 7 8 9

17. A 66 year old man had a heart attack three years ago. He feels fine and wants to know if he should have a stress test to check on his heart.

Bad Idea | Okay Idea | Good Idea

1 2 3 4 5 6 7 8 9

**18. In your opinion, what percent of medical spending is wasted?**

1%

5%

10%

30%

**19. How much money do you think the US spends on unnecessary health care every year?**

1 billion dollars

10 billion dollars

100 billion dollars

200 billion dollars

**20. How much do you think a nuclear stress test costs?**

Less than $100

$100-$500

$500-$1,000

More than $1,000

**21. Have you ever had a nuclear stress test in the past?**

Yes

No

**22. If yes, what was it like? Please describe.**

________________________________________________________________

________________________________________________________________

________________________________________________________________

**23. Which of these would be a good enough reason to get a nuclear stress test for someone who feels well and has no symptoms (no chest pain, no trouble breathing, good energy level)? Circle Yes or No for each one.**

Diabetes (Yes / No)

Abnormal electrocardiogram (Yes / No)

Family history of heart disease (Yes / No)

Before taking drugs to improve a man’s erection (Yes / No)

High risk of heart disease (Yes / No)

Annual checkup (Yes / No)

Before having a low-risk surgery (Yes / No)

History of heart attack a year ago (Yes / No)

History of stent a year ago (Yes / No)

**24. Have you heard of the Choosing Wisely campaign?**

Yes

No

**25. If so, what do you know about it?**

________________________________________________________________

________________________________________________________________

________________________________________________________________

________________________________________________________________

**26. Have you ever specifically told your doctor you wanted a specific test done such as a blood test, stress test, or X ray?**

Yes

No

**27. If you answered yes to question 26, what tests have you asked for?**

________________________________________________________________

**28. If you answered yes to question 26, what reasons made you want to order the tests? (circle all that apply)**

Family history of a disease

Close friend with a recent illness

Risk factors (such as smoking)

Heard about test on television or internet

Recommended by a friend or family member

Other reason:____________________________________________________

________________________________________________________________

**29. If you answered yes to question 26, how does your doctor usually respond when you ask for a test to be done?**

Orders the test

Discusses the test with you before deciding

Refuses to order the test

**30. Which of these things expose you to radiation? (circle all that apply)**

CAT scan of the head

Television and computer monitors

Airport scanner

Nuclear Stress test

“Blacklight” bulb

Microwave

**31. What has the most radiation exposure? (circle only one)**

CAT scan of the head

Television and computer monitors

Airport scanner

Nuclear Stress test

“Blacklight” bulb

Microwave

**32. How much do you think radiation from a nuclear stress test increases the risk of cancer?**

No increase

Slight increase

Mild increase

Serious increase

**33. Is it ever a bad idea to get a nuclear stress test?**

Yes

No

**34. Please explain:**

_____________________________________________________________________________

_____________________________________________________________________________

_____________________________________________________________________________

_____________________________________________________________________________

**35. Are you confident that your answers to this survey will remain anonymous?**

Yes

No
