# Supplementary material for: Perceptions of patients and providers on myocardial perfusion imaging for asymptomatic patients, choosing wisely, and professional liability
Source: BMC Health Serv Res. 2017 Aug 11;17:553. doi: 10.1186/s12913-017-2510-y (PMC5553740; doi:10.1186/s12913-017-2510-y)
Supplement: Supplementary file 2 — Provider Survey. Description: Full version of questionnaire given to providers. (DOCX 27 kb) [file 12913_2017_2510_MOESM2_ESM.docx]

**1. Please indicate your professional training**

MD or DO

ARNP or PA

Other: __________________________________

**2. Please indicate your level of clinical experience**

Still in training (ie: resident, fellow, etc.)

Completed training less than 5 years ago

Completed training 5 to 15 years ago

Completed training more than 15 years ago

**3. Please indicate your primary area of clinical practice**

Primary care (Internal Medicine/Family Medicine/Geriatrics)

Hospitalist Medicine

Cardiology

Other: _______________________

**4. Primary workplace:**

University of Florida

Private Practice

VA (5/8 or greater)

Other academic institution

**5. Please select the primary setting for your patient interactions**

Inpatient

Outpatient, based in a medical center

Outpatient, based in a rural or outlying clinic

**6. Please estimate how frequently you (or one of your trainees) order nuclear stress tests in your practice or while you are on service or in clinic:**

Never

Once a month or less

Once a week or less

Once a day or less

More than once per day

**7. What is the most common indication for which you order nuclear stress tests?**

Screening for heart disease in asymptomatic patients

Testing for heart disease in patients with symptoms

Pre-operative risk assessment

Assess for ischemia in patients with known heart disease

Other: ____________________________________________________________

**For each of the following scenarios, please indicate if you consider a NUCLEAR STRESS TEST to be appropriate, inappropriate, or uncertain. PLEASE NOTE, this is not intended to ask if this is the *best test* or the *first test* that you would choose for the patient, but only whether a nuclear stress test would be appropriate.**

8. A 62 year old woman had an NSTEMI 2 years ago treated with a drug-eluting stent. Over the past several weeks she has noted chest discomfort when walking with her friends. She also has hypertension, hyperlipidemia, and non-insulin-dependent diabetes, which are controlled with medications.

Inappropriate | Uncertain | Appropriate

1 2 3 4 5 6 7 8 9

9. A 45 year old woman presents with chest discomfort that occurs sporadically, sometimes when she is walking while carrying groceries in from the store and lasting a few seconds. She has no history of heart disease and her ECG does not show any changes in the ST-T segments.

Inappropriate | Uncertain | Appropriate

1 2 3 4 5 6 7 8 9

10. A 52 year old man is concerned because his father died at 52 of a myocardial infarction. He has no cardiac symptoms with exertion and a 50 pack-year smoking history. Blood pressure (SBP 120), lipids (Tot Chol 160, HDL 40), and ECG are unremarkable.

Inappropriate | Uncertain | Appropriate

1 2 3 4 5 6 7 8 9

11. A 58 year old woman complains of chest pain. She last noted the symptoms while climbing stairs, but has also had similar symptoms while watching television. The symptoms are not so severe that she has to stop climbing, but she thinks they sometimes improve when she rests. Her ECG has first degree AV block.

Inappropriate | Uncertain | Appropriate

1 2 3 4 5 6 7 8 9

12. A 66 year old man is a new patient establishing care. He suffered a myocardial infarction about three years ago successfully treated with a stent to the LAD artery. Report from that procedure indicates mild blockages in the other arteries. He is on appropriate medical therapy, his blood pressure is controlled, and he has no symptoms.

Inappropriate | Uncertain | Appropriate

1 2 3 4 5 6 7 8 9

**13. How often do you order nuclear stress tests in patients without any symptoms?**

Never

Rarely

Sometimes

Often

**14. Guidelines for heart disease prevention and management recommend to routinely perform screening tests for patients without symptoms.**

True

False

**15. What is the benefit for ordering nuclear stress tests in asymptomatic patients?**

Research clearly shows patient benefit

Evidence is mixed, but overall shows patient benefit

Evidence is mixed, but overall shows no patient benefit

Research clearly shows no patient benefit

No good evidence exists, recommendations are based on expert opinion

**16. Of the following factors, which would be sufficient to justify a nuclear stress test for a patient without symptoms? (Circle Yes or No for each)**

Diabetes (Yes / No)

Abnormal ECG (Yes / No)

Family history of heart disease (Yes / No)

Prior to prescribing drugs for erectile dysfunction (Yes / No)

High CHD risk (ie: Framingham) (Yes / No)

Annual evaluation (Yes / No)

Pre-op for low-risk surgery (Yes / No)

History of myocardial infarction a year ago (Yes / No)

History of stent a year ago (Yes / No)

**17. How worried are you that patients will have bad outcomes if they do not have stress testing?**

Not worried

Slightly worried

Somewhat worried

Very worried

**18. How confident are you in evaluating patients with complaints of chest pain or other symptoms possibly due to heart disease?**

Very confident

Somewhat confident

Not too confident

Not at all confident

**19. What percent of medical spending is wasted?**

1%

5%

10%

30%

**20. How much money does the US spend on unnecessary health care every year?**

1 billion dollars

10 billion dollars

100 billion dollars

200 billion dollars

**21. How much do you think a nuclear stress test costs?**

Less than $100

$100-$500

$500-$1,000

More than $1,000

**22. What has the most radiation exposure?**

CAT scan of the head

Television and computer monitors

Airport scanner

Nuclear Stress test

“Blacklight” bulb

Microwave

**23. How much does radiation from a nuclear stress test increase the risk of cancer?**

No increase

Slight increase

Mild increase

Significant increase

**24. Have you heard of the Choosing Wisely campaign?**

Yes

No

**25. If so, what do you know about it? Please describe.**

_____________________________________________________________________

_____________________________________________________________________

_____________________________________________________________________

**26. How often do patients ask for a test or procedure that you think is unnecessary?**

Every day

Almost every day

Several times a week

About once a week

At least once a month

Less often

**27. When patients ask for an unnecessary test or procedure, how often do you talk to them about why they should not have the test or procedure?**

Always

Almost always

Frequently

Not too often

Rarely or never

**28. How comfortable do you feel talking to patients about why they should avoid an unnecessary test or procedure?**

Very comfortable

Somewhat comfortable

Not too comfortable

Not at all comfortable

**29. Have you ever ordered a nuclear stress test because a patient insisted or felt that they needed it?**

Yes

No

**30. In your opinion, do recommendations from professional societies regarding when to order tests (such as appropriate use criteria) improve any of the following? (circle all that apply)**

Quality of care

Safety of care

Health of populations of patients

Lower overall costs of care

Decision making

Education of ordering physicians

**31. What is an acceptable rate of inappropriate procedures in a population?**

0-5%

5-10%

10-20%

20-30%

>30%

**32. In general, how worried are you about medical malpractice?**

Not worried

Slightly worried

Somewhat worried

Very worried

**33. To what extent do you feel that your employment situation protects you from medical malpractice lawsuits?**

No protection

Some protection

Good protection

Basically immune from lawsuits

**34. Have you ever ordered a nuclear stress test because you were worried about the potential of a malpractice lawsuit if you failed to order the test?**

Yes

No

**35. If you wanted to learn more when nuclear stress tests are unnecessary, which of the following would be helpful? (circle all that apply)**

In-person lectures on appropriate use

Materials on appropriate use that you can review at your own pace

Monthly feedback about appropriateness of tests you order

Real-time feedback about appropriateness of tests you order

A computer system to effectively guide appropriateness at the time you order a test

**36. Are you familiar with Appropriate Use Criteria (AUC) for nuclear stress tests?**

I had never heard of the AUC prior to this survey

I have am aware of the AUC, but have not read them/do not use them

I have am aware of the AUC and use them occasionally in decision making

I have am aware of the AUC and use them routinely in decision making

**36. Would you be interested in using any of the following to help improve the appropriate use of tests? (check all that apply)**

Smartphone app

Pocket card or paper reminders

Assistance built into your electronic ordering system

Other: ________________________

**37. What reasons do you think are inappropriate for ordering a nuclear stress test?**

_____________________________________________________________________

_____________________________________________________________________

_____________________________________________________________________

_____________________________________________________________________

**38. Are you confident that your answers to this survey will remain anonymous?**

Yes

No

**For VA providers only**

When ordering nuclear stress tests, the CPRS system requires you to complete screening questions to determine if the test is needed. Please rate your agreement with each of the following statements.

**1. It is difficult to find the order for a nuclear stress test.**

Strongly Agree

Agree

Disagree

Strongly Disagree

**2. The screening questions are helpful for decision making.**

Strongly Agree

Agree

Disagree

Strongly Disagree

**3. The screening questions frequently do not apply to my patient.**

Strongly Agree

Agree

Disagree

Strongly Disagree

**4. Sometimes I need to bypass the screening questions to order what I think is best for my patient.**

Strongly Agree

Agree

Disagree

Strongly Disagree
